# Prevalence of human papillomavirus genotypes in women of different ethnicity from rural northwestern Ecuador

**DOI:** 10.1186/s44263-024-00078-x

**Published:** 2024-07-10

**Authors:** Rosa de los Ángeles Bayas-Rea, Karina Ponce, Andrea Guenther, Juan D. Mosquera, Carolina Armijos, Lorena Mejía, Gabriela Bustamante, Sonia Zapata

**Affiliations:** 1 Instituto de Microbiología, Colegio de Ciencias Biológicas y Ambientales, Universidad San Francisco de Quito USFQ, Quito, 170901 Ecuador; 2 Departamento de Ciencias de La Vida y de La Agricultura, Universidad de Las Fuerzas Armadas, Sangolquí, Ecuador; 3 Department of Anaesthesiology, Klinikum Bremen-Ost, Bremen, Germany; 4 Laboratorio de Biotecnología Vegetal, Colegio de Ciencias Biológicas y Ambientales, Universidad San Francisco de Quito USFQ, Quito, 170901 Ecuador; 5 Instituto de Medicina Social y Desafíos Globales, Escuela de Salud Pública, Colegio de Ciencias de La Salud, Universidad San Francisco de Quito USFQ, Quito, 170901 Ecuador

**Keywords:** Esmeraldas, Ethnic group, High-risk HPV, Remote communities, Variants

## Abstract

**Background:**

In Ecuador, cervical cancer is the third most common cancer among women and the second most common cause of cancer-related death in women. Although HPV represents a serious public health problem worldwide, the information about its prevalence and genotypes in remote communities of Ecuador is limited. The aim of this study was to determine the distribution of HPV genotypes among ethnic minority women from two remote communities of the northwestern region of Ecuador: Afro-Ecuadorians and Chachis (Amerindian group).

**Methods:**

We included 291 women who responded to a short survey and were screened for HPV by the amplification of the L1 gene and a nested multiplex PCR to detect 14 high risk (HR) genotypes. A survey collected information on ethnicity, age, community access, and sexual and gynecological history. We identified risk factors associated with HPV infection and co-infection using multivariate logistic regression to calculate odds ratio (OR) and Wald 95% confidence intervals (CI).

**Results:**

Overall, HPV prevalence in the study cohort was 56%, and the five most common HR-HPV genotypes were HPV-58, -16, -68, -39, and -43; however, the distribution of HPV genotypes varied according to ethnicity. We identified lineage A (European variant) for HPV-16 and sublineage A2 for HPV-58 in both ethnic groups. Adjusting for ethnicity, age, community access, and number of sexual partners, we found that Afro-Ecuadorian women were less likely to have an HPV infection than Chachi women (*OR*: 0.49, 95% *CI*: 0.25, 0.96), and that participants in communities only accessible by river had 64% less chances of an HPV infection when compared to women in communities accessible by road (*OR*: 0.36, 95% *CI*: 0.19, 0.71), and women with 2 to 3 sexual partners had 2.47 times the odds of HPV infection than participants with 0–1 partners (*OR*: 2.47, 95% *CI*: 1.32, 4.6). Similar associations were observed with prevalence of co-infection.

**Conclusions:**

This study provides baseline knowledge regarding the prevalence and distribution of HPV genotypes in ethnic groups of the northwestern coastal Ecuador and essential information for the implementation of appropriate HPV testing and vaccination program to prevent cervical cancer.

## Background

Cervical cancer is the fourth most frequently diagnosed cancer and cause of death among women in low- and middle-income countries, with an estimated 604,127 new cases and 341,831 deaths in 2020 [1]. In Ecuador, cervical cancer is the third cause of cancer and the second most common cause of death among young women of ages 15 to 44 [2]. Annually, an estimated of 1534 new cervical cancer cases and 813 cervical cancer deaths occur in the country [3]. The persistent infection with oncogenic genotypes of human papillomavirus (HPV), named high-risk (HR) genotypes, is the most important cause of cervical cancer [2, 4].

The six most common HR-HPV genotypes worldwide are HPV-16, HPV-18, HPV-45, HPV-52, HPV-31, and HPV-58 [5–7]. However, the distribution of HPV genotypes and its prevalence vary broadly throughout the world [4, 8]. In many regions, HPV-16 and -18 are the most prevalent oncogenic genotypes, responsible for about 70% of all cervical cancer [8–10]. In East Asia, some parts of Europe, Latin America, and Africa, HPV-58, HPV-52, HPV-31, and HPV-45 are among the most common genotypes [5, 7, 10, 11].

HPV genotypes can be further classified into lineages and sublineages (variants). These variants show distinct association with persistence, high viral load, progression to precancer, and cancer, suggesting that each lineage has a distinct evolutionary history [12–14]. It has also been suggested that host genetic factors (human leukocyte antigen, HLA, haplotypes) may influence the progression to cancer of some HPV variants [15]. For instance, a study conducted by Mejia et al. [16] in Ecuadorian women showed that HPV-16 from the European lineage was the most common type. The European lineage is considered one of the least carcinogenic.

Women from rural communities in low- and middle-income countries are more vulnerable to develop cervical cancer mainly due to limited access to prevention and treatment. Studies have revealed a disproportionate burden of HPV-related diseases in these populations due to limited access to healthcare, cultural barriers, and socioeconomic disparities [17, 18]. A few studies have estimated HPV prevalence, genotype distribution, and risk factors in rural or indigenous populations in South American countries [19–22]. Namely, a recent study in the southern highlands of Ecuador concluded that indigenous women had a higher prevalence of HPV infection compared to mestizos (a person of mixed race, especially one having Spanish and indigenous descent) [23]. In Ecuador, recent studies focused on HPV and its clinical manifestations in indigenous populations of the Andean and high Andean region [24, 25] but have not included communities in remote coastal areas. The aim of our study was to determine the main HPV genotypes and oncogenic variants among women from rural communities in the northwestern coastal region of Ecuador and identify risk factors associated with HPV infection in this population.

## Methods

### Study design and participant selection

We carried out a cross-sectional observational study in the northwestern coastal region of Ecuador, in canton Eloy Alfaro, Esmeraldas Province, between June 2006 and October 2008. Women aged 14 to 75 years from 20 remote communities participated in the study. According to the 2010 census, this region was mainly inhabited by Afro-Ecuadorians (43.9%; 234,467 people) and Mestizos (44.7%; 238,739), with a smaller proportion of indigenous Amerindians (2.8%; 10,222 Chachi people) and 8.3% other ethnic/racial identities [26, 27].

The sample size was based on previous studies in Ecuador and data from the Ministry of Public Health estimating the prevalence of HPV infection to range between 40 and 80%. To estimate prevalence with 95% confidence and a 5% margin of error, we projected a need for approximately 245 to 360 patients [28].

We employed convenience sampling to recruit female patients who were receiving gynecologist care at Hospital General de Borbón. Participants were also invited by a gynecologist in a medical expedition aimed at accessing remote communities. Eligible participants were women who had not undergone a Pap smear in the previous year or had never undergone one, were not pregnant, were not actively menstruating, and had not undergone a hysterectomy.

### Data collection

A trained team member interviewed participants with a short survey regarding sociodemographic and sexual history information. In conjunction with community leaders, we opted to use interviewers to facilitate survey completion among all participants. This allowed us to respect cultural oral traditions from the Chachis and unify data collection strategies across different literacy levels in the communities. Ethnicity was self-reported, and age in years was reported at the time of the survey and divided into four categories for analysis (under 25, 25 to 34, 35 to 44, and 45 +). Additionally, a survey collected data on community access (only road, only by river, both road and river), number of life sexual partners (0, 1, 2 to 3, 4 or more), start of sexual activity (< 15 years, 15–18, > 18), number of pregnancies (1 to 3, 4 to 6, 7 +), any gynecological symptoms reported at the time of the survey (yes/no), and current birth control use (none/any). We decided to omit educational data due to the prevalent lack of education or limited primary education among participants.

### Sample collection

We collected a total of 291 samples of ecto- and endocervix using a cytobrush for HPV DNA extraction. We stored the samples in sterile phosphate buffered saline solution (PBS) 1X (37-mM NaCl, 2.7-mM KCl, 4.3-mM Na_2_HPO_4_.7H_2_O, 1.4-mM KH_2_PO_4_, pH 7.0) at − 20 °C until molecular analysis. The time elapsed between sample collection and processing was 2 weeks. We conducted DNA extraction at the Instituto de Microbiología of the Universidad San Francisco de Quito.

### DNA extraction and HPV genotyping

For DNA extraction, we used a modified cetyl trimethyl ammonium bromide (CTAB) protocol [19]. We released the cervical cells from the cytobrush by agitation and washed twice in 1X PBS solution, pH 7. We lysed the pelleted cells with 700-μl lysis buffer (2% CTAB weigh/vol, 1.4-M NaCl, 20-mM EDTA pH 8, 100-mM Tris–HCl, pH 8) and incubated for 2 h at 65 °C with homogenization each 30 min. We used chloroform:alcohol-isoamyl (24:1) for the extraction of DNA followed by precipitation with 100% ethanol and sodium acetate 3 M (pH 5). Finally, we resuspended the DNA in 50 μl of Tris–EDTA buffer (TE; 10-mM Tris–HCl, pH 8, 0.1-mM EDTA) and stored it at − 20 °C [19]. We determined the quantity and quality of the genomic DNA using a NanoDrop™ 1000 Spectrophotometer (Thermo Scientific) according to the manufacturer’s instructions.

We used the β-actin partial gene as internal control to analyze the quality of template DNA. We amplified a fragment of 290 bp by PCR according to du Breuil et al. [29]. For HPV genotyping, we considered only those samples successfully amplified.

We carried out the detection of HPV by amplification of the L1 gene, as described elsewhere [30], with minor changes. Briefly, we prepared amplification reactions in 20-μl reaction mixture containing a 1X reaction buffer, 1.5-mM MgCl2, 200-μM of each dNTP, 0.25-μM of primers MY09 (5′CGTCCMARRGGAWACTGATC3′) and MY11 (5′GCMCAGGGWCATAAYAATGG3′), 1 U of GoTaq DNA Polymerase (Promega Corporation, Madison), and 20 ng of DNA. The amplification program had an initial denaturation of 94 °C for 3 min, followed by 40 cycles of 1 min each (denaturation at 94 °C, annealing at 55 °C, and elongation at 72 °C). The final extension step was done at 72 °C for 10 min. We analyzed the PCR products on 1.5% agarose gels, visualized by ethidium bromide staining under UV light exposure. We used DNAse-free water as negative control and an HPV-infected sample as positive control.

We performed the HPV genotyping using a nested multiplex PCR described previously by Sotlar et al. [30]. First, we carried out a PCR with a mix of three degenerate consensus primers: GP-E6-3F (5′-GGGWGKKACTGAAATCGGT-3′), GP-E6-5B (5′-CTGAGCTGTCARNTAATTGCTCA-3′), GP-E6-6B (5′-TCCTCTGAGTYGYCTAATTGCTC-3′). We amplified a fragment of 630 bp of the viral E6-E7 oncogenes. We carried out E6/E7 PCR amplification reactions in 20-μl reaction mixture containing 1X PCR buffer, 3-mM MgCl_2_, 200 μM of each dNTP, 0.25 μM of each primer (E6/E7), 0.7 U GoTaq DNA polymerase (Promega Corporation, Madison), and 200 ng of DNA. PCR conditions consisted of an initial denaturation step at 92 °C for 2 min, then 36 cycles of denaturation at 92 °C for 50 s, annealing at 45 °C for 50 s, and extension at 72 °C for 50 s, followed by a final extension step at 72 °C for 10 min. After that, we carried out a multiplex PCR with four cocktails, each one containing four or five different primers pairs. We used a total of 18 pairs of genotype-specific internal nested PCR primers in a multiplex cocktail for high-risk genotypes: 16, 18, 31, 33, 35, 39, 45, 51, 52, 56, 58, 59, 66, and 68 and low-risk genotypes: 6/11, 42, 43, and 44 [30]. We performed nested PCR amplification reactions in 20-μl reaction mixture containing 1X PCR buffer, 2.5-mM MgCl_2_, 200 μM of each dNTP, 0.75 μM of each primer, 0.7 U of *Taq* DNA polymerase (Promega Corporation, Madison), and 2 μl of E6/E7 amplicon. PCR conditions consisted of an initial denaturation step at 94 °C for 4 min, followed by 35 cycles of denaturation at 92 °C for 30 s, annealing at 56 °C for 30 s, and extension at 72 °C for 45 s, followed by a final extension step at 72 °C for 10 min. We analyzed the PCR products on 1.5% agarose gels, visualized by ethidium bromide staining under UV light exposure, and finally photographed. We used a reaction mixture with PCR water as negative control. We delivered results of HPV genotyping to the local healthcare center.

### Variant analysis

We chose all mono-infected samples with the most common genotypes (HPV-16 and 58) for variant analyses. We purified the DNA fragments of gene L1 from agarose gels with PureLink Quick Gel Extraction and PCR Purification Combo (Invitrogen, Carlsbad) according to the manufacturer’s instructions. We sequenced the purified samples at Functional Biosciences (https://functionalbio.com/web/) and submitted them to GenBank (Accession numbers MK303335-MK303343). We aligned each gene nucleotide sequence independently with HPV-16 and 58 reference variants from GenBank using MEGA software version X [31]. For phylogenetic analyses, we included other Ecuadorian HPV-16 and 58 L1 gene sequences submitted to GenBank (Accession numbers KP794892-KP794894, KP794914, KP794917, and KP794918) by Mejía et al. [16]. We used representative genomes for HPV-16 (termed A, B) and 58 lineages (termed A, C) and sub-linages (A1-A4/B2 for HPV-16 and A1-A3 for HPV-58) as variant references [13]. Evolutionary history was inferred using the maximum likelihood method and Tamura 3-parameter model [32]. The percentage of trees in which the associated taxa clustered together is shown next to the branches. Initial tree(s) for the heuristic search were obtained automatically by applying neighbor-join and BioNJ algorithms to a matrix of pairwise distances estimated using the maximum composite likelihood (MCL) approach and then selecting the topology with superior log-likelihood value. Evolutionary analyses were conducted in MEGA X [31].

### Statistical analyses

We summarized demographic characteristics using frequency tables. Prevalence of HPV infection was coded as positive if the presence of any of the tested genotypes was detected. If a single genotype was detected, the case was deemed a mono-infection, and if more than one genotype was detected, it was deemed a co-infection. We estimated the prevalence of overall HPV infection, co-infection, and by each specific genotype in the general sample, as well as stratified by ethnic group.

To identify risk factors associated with HPV infection and co-infection, we used univariate and multivariate logistic regression to calculate odds ratio (OR) and Wald 95% confidence intervals (CI). All statistical analysis was conducted in Stata 16.1 [33].

## Results

A total of 291 women participated in the study, aged 14 to 75 years with a median age of 33 years. Most of them identified as Afro-Ecuadorian (73.5%), and 60.5% lived in communities only accessible by river (Table 1). Most participants (59.5%) reported having 1 life sexual partner, with 24.1% mentioning they had started sexual activity between 15 and 18 years of age, but over 61% had missing information on this variable.

**Table 1:** Characteristics of the study sample

Characteristic	*N*	%
**Ethnicity**		
Chachi	77	26.5
Afro-Ecuadorian	214	73.5
**Age**		
Under 25	63	21.7
25 to 34	85	29.2
35 to 44	64	22.0
45 +	79	27.2
**Community access**		
Only road access	72	24.7
Only river access	176	60.5
Both road and river	43	14.8
**Number of life sexual partners**		
0	6	2.1
1	173	59.5
2 to 3	81	27.8
4 or more	16	5.5
Missing information	15	5.2
**Start of sexual activity**		
< 15	19	6.5
15–18	70	24.1
> 18	24	8.3
Missing information	178	61.2
**Number of pregnancies**		
1 to 3	36	12.4
4 to 6	44	15.1
7 +	42	14.4
Missing information	169	58.1
**Any symptom reported**		
No	200	68.7
Yes	91	31.3
**Current birth control use**		
None	24	8.3
Some	16	5.5
Missing information	251	86.3

As shown in Table 2, among the 291 women included in the study, 56% were positive for HPV (57% among Afro-Ecuadorian women and 53% among Chachi women). Mono-infections were more common than co-infections in both Afro-Ecuadorian (76%) and Chachi women (79%). Among 163 positive HPV samples, the most common genotype was HPV-58 (37% of all positive samples) followed by HPV-16 (28%) and HPV-68 (13%). Low-risk genotypes 6/11 were present in 20 of the positive samples representing 12% of the HPV cases, but only 4 of these were present as mono-infections; the remaining 16 were present as co-infections with high-risk genotypes. Oncogenic type HPV-18 was only identified in 1% of the positive samples (Table 2).

**Table 2:** HPV prevalence by ethnic group in rural communities of northwestern Ecuador, including mono-infection and coinfection and HPV risk genotype

	**Chachi**		**Afro-Ecuadorian**		**Total**	
	***N***	**Col %**	***N***	**Col %**	***N***	**Col %**
**Any HPV infection**						
Yes	41	53.3	122	57.0	163	56.0
No	36	46.8	92	43.0	128	44.0
**Co- or Mono-infection**						
Co-infection	16	20.8	52	24.3	68	23.4
Mono-infection	61	79.2	162	75.7	223	76.6
**Genotypes**						
High risk 16 or 18	4	5.2	44	20.6	48	16.5
Other high risk	36	46.8	75	35.1	111	38.1
Only low risk (6 or 11) infection	1	1.3	3	1.4	4	1.4
**HPV genotypes (mono- or co-infection)**						
g58	23	29.9	37	17.3	60	20.6
g16	4	5.2	42	19.6	46	15.8
g68	4	5.2	18	8.4	22	7.6
g39	0	0.0	20	9.4	20	6.9
g6_11	7	9.1	13	6.1	20	6.9
g43	8	10.4	10	4.7	18	6.2
g52	6	7.8	11	5.1	17	5.8
g35	0	0.0	16	7.5	16	5.5
g42	3	3.9	9	4.2	12	4.1
g44	2	2.6	8	3.7	10	3.4
g45	3	3.9	6	2.8	9	3.1
g51	4	5.2	5	2.3	9	3.1
g66	1	1.3	8	3.7	9	3.1
g56	2	2.6	4	1.9	6	2.1
g33	0	0.0	3	1.4	3	1.0
g18	0	0.0	2	0.9	2	0.7
g31	2	2.6	0	0.0	2	0.7
g59	0	0.0	1	0.5	1	0.3

*Abbreviations: HPV* human papillomavirus, *col* column

**Fig. 1:**
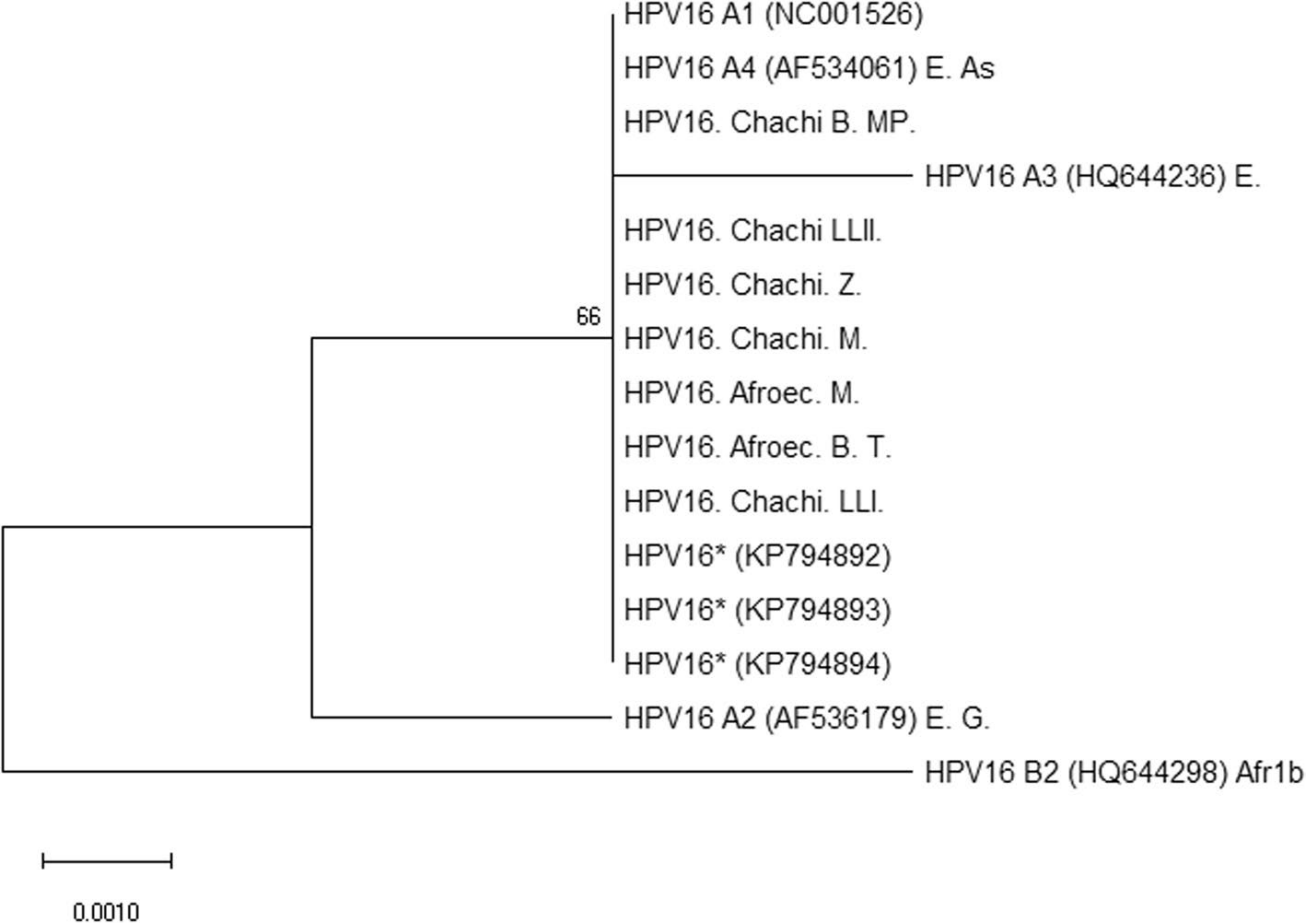
Maximum-likelihood tree using nucleotide sequences of L1 genes from HPV-16 mono-infected samples obtained in this study (MK303335-MK303341). Sequences reported here are denoted as Chachi, and Afroec for Afro-Ecuadorian, followed by the initials of the names of the locations. B, Borbon; MP, Mata de Plátano; LLI, Loma Linda I; LLII, Loma Linda II; Z, Zapallo; M, Maldonado; T, Tigüinza. L1 sequences from Mejía et al. [16] (KP794892-KP794894) were included in the analysis. Representative genomes for HPV-16 lineages (termed A, B) and sublineages (termed A1, A2, A3, A4/B2) were used as variant references [13]. Regional variants are indicated as follows: E, European variant; As, East Asian; G, German; Afr, African

**Fig. 2:**
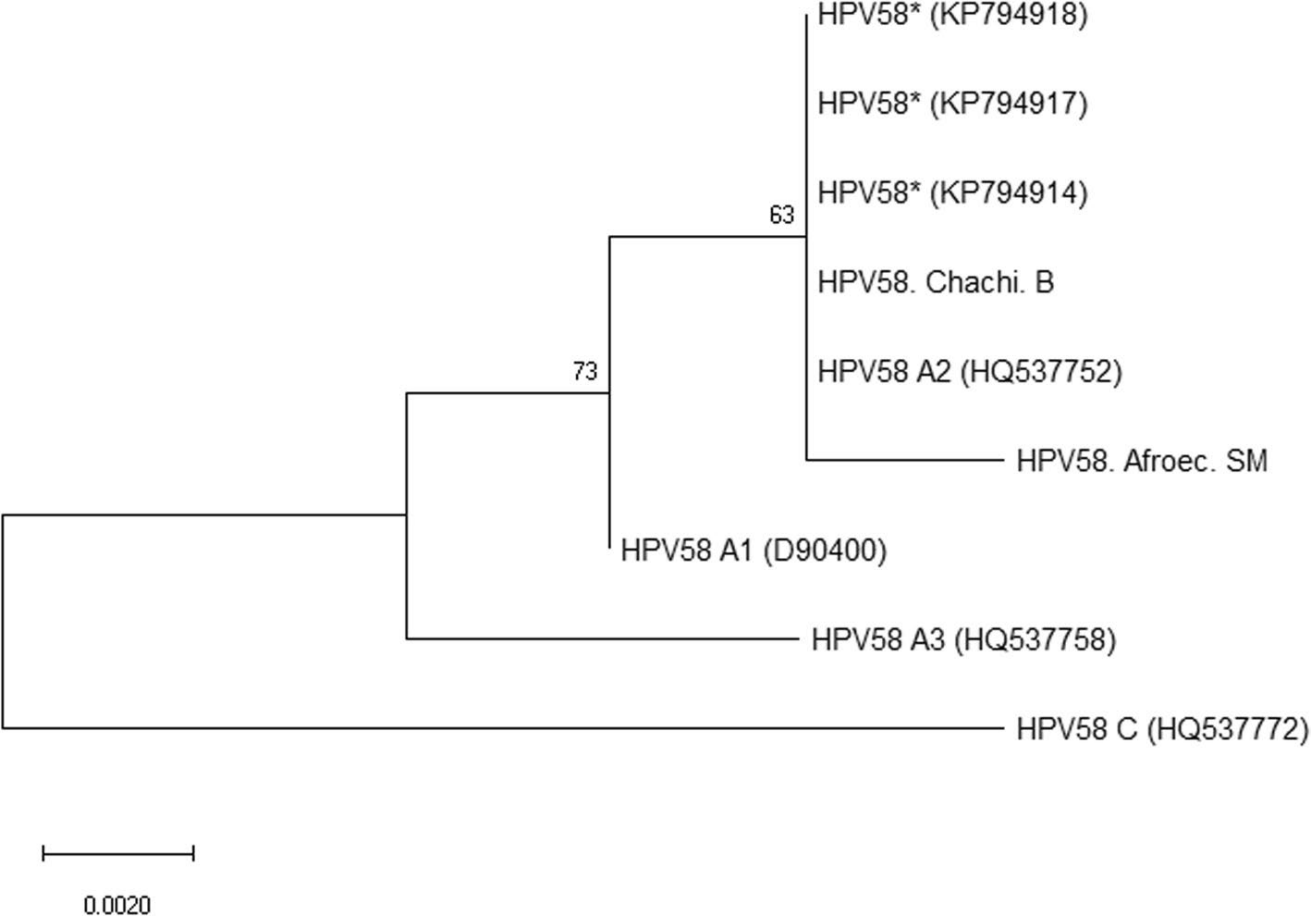
Maximum-likelihood tree using nucleotide sequences of L1 genes from HPV-58 mono-infected samples obtained in this study (MK303342 and MK303343). Sequences reported here are denoted as Chachi, and Afroec for Afro-Ecuadorian, followed by the initials of the name of the locations. B, Borbon; SM, San Miguel. L1 sequences from Mejía et al. [16] (KP794914, KP794917, and KP794918) were also included in the analysis. Representative genomes for HPV-58 lineages (termed A, C) and sublineages (termed A1, A2, A3) were used as variant references [13]

A total of nine positive samples from mono-infections of HPV-16 (seven) and HPV-58 (two) were used for the amplification and sequencing of the L1 gene. The sequences’ length mean was 351 bp (range 291–422). All nucleotide sequences from HPV-16 amplicons (seven samples) grouped into the European branch (lineage A; Fig. 1), independently of their ethnic origin. The sublineage could not be identified since all sequences grouped into A1 (NC001526) and A4 variants with no characteristic SNPs to differentiate them. Regarding HPV-58, the two sequences (one Chachi sample and one from an Afro-Ecuadorian woman) pooled into the A2 sublineage (Fig. 2); nevertheless, the Afro-Ecuadorian sample showed a SNP in G6641A of gene L1.

Based on univariate (crude) logistic regression, we identified that community access and number of life sexual partners were associated to prevalence of HPV infection, while ethnicity, age, start of sexual activity, and whether symptoms were reported at the time of the survey were not (Table 3). Specifically, women from communities only accessible by river had 51% less chance of HPV infection than women in communities accessible by road (*OR*: 0.49, 95% *CI*: 0.28, 0.86). On the other hand, participants with 2 to 3 life partners were 1.83 (95% *CI*: 1.06, 3.15) times more likely to be HPV positive than participants with 0 or 1 life sexual partners. When we adjusted for ethnicity, age, road access, and number of sexual partners, we found that Afro-Ecuadorian women were less likely to have an HPV infection than Chachi women (*OR*: 0.49, 95% *CI*: 0.25, 0.96), and that participants in communities only accessible by river had 64% less chances of an HPV infection when compared to women in communities accessible by road (*OR*: 0.36, 95% *CI*: 0.19, 0.71), and women with 2 to 3 sexual partners had 2.47 times the odds of HPV infection than participants with 0–1 partners (*OR*: 2.47, 95% *CI*: 1.32, 4.6).

**Table 3:** Associations between demographic factors and HPV infection among 291 women from rural communities in northwestern Ecuador

	**Any HPV infection**	**Univariate logistic regression**			**Multivariate logistic regression**		
	***N***	**OR**	**95% *CI***	***p*-value**	**OR**	**95% *CI***	***p*-value**
**Ethnicity**							
Chachi	41	*Ref*			*Ref*		
Afro-Ecuadorian	122	1.16	(0.69, 1.96)	0.57	0.49	(0.25, 0.96)	0.04
**Age, in years**							
Under 25	38	*Ref*			*Ref*		
25 to 34	50	0.94	(0.48, 1.83)	0.86	0.82	(0.41, 1.66)	0.58
35 to 44	35	0.79	(0.39, 1.61)	0.52	0.72	(0.33, 1.55)	0.40
45 +	40	0.67	(0.35, 1.32)	0.25	0.68	(0.33, 1.40)	0.29
**Community access**							
Only road access	47	*Ref*			*Ref*		
Only river access	84	0.49	(0.28, 0.86)	0.01	0.36	(0.19, 0.71)	0.00
Both road and river	32	1.55	(0.67, 3.58)	0.31	1.58	(0.64, 3.89)	0.32
**Number of life sexual partners**							
0 or 1	91	*Ref*			*Ref*		
2 to 3	53	1.83	(1.06, 3.15)	0.03	2.47	(1.32, 4.60)	0.01
4 or more	12	2.90	(0.90, 9.34)	0.07	2.67	(0.78, 9.10)	0.12
**Start of sexual activity**							
< 15	12	0.71	(0.19, 2.54)	0.41			
15–18	43	0.65	(0.24, 1.79)	0.89			
> 18	17	*Ref*					
**Any symptom reported**							
No	115	*Ref*					
Yes	48	0.83	(0.50, 1.36)	0.45			

Odds ratios (OR) and 95% confidence intervals (CI) were calculated using univariate and multivariate logistic regression

Similarly, univariate (crude) logistic regression models in Table 4 identified number of life sexual partners and age of start of sexual activity as factors associated with co-infection. Specifically, women with 2 to 3 life partners were 2.39 (95% *CI*: 1.31, 4.36) times more likely to have an HPV co-infection than participants with 0 or 1 life sexual partners. When we adjusted for ethnicity, age, road access, and number of sexual partners, we found that women with 2 to 3 sexual partners had 3.48 times the odds of HPV infection than participants with 0–1 partners (*OR*: 3.48, 95% *CI*: 1.72, 7.02), and that women with 4 or more sexual partners had 3.58 times the odds of HPV infection than participants with 0–1 partners (*OR*: 3.58, 95% *CI*: 1.15, 11.1).

**Table 4:** Associations between demographic factors and HPV co-infection among 291 women from rural communities in northwestern Ecuador

	**Co-infection**	**Univariate logistic regression**			**Multivariate logistic regression**		
	***N***	**OR**	**95% *CI***	***p*-value**	**OR**	**95% *CI***	***p*-value**
**Ethnicity**							
Chachi	16	*Ref*			Ref		
Afro-Ecuadorian	52	1.22	(0.65, 2.30)	0.53	0.43	(0.19, 1.00)	0.05
**Age**							
Under 25	18	*Ref*			Ref		
25 to 34	23	0.93	(0.45, 1.92)	0.84	0.71	(0.33, 1.56)	0.40
35 to 44	11	0.52	(0.22, 1.21)	0.13	0.36	(0.14, 0.92)	0.03
45 +	16	0.63	(0.29, 1.38)	0.25	0.65	(0.28, 1.51)	0.32
**Community access**							
Only road access	20	*Ref*			Ref		
Only river access	33	0.60	(0.32, 1.14)	0.12	0.47	(0.22, 1.02)	0.06
Both road and river	15	1.39	(0.62, 3.14)	0.42	1.59	(0.64, 3.94)	0.73
**Number of life sexual partners**							
0 or 1	31	*Ref*			Ref		
2 to 3	27	2.39	(1.31, 4.36)	0.01	3.48	(1.72, 7.02)	0.001
4 or more	7	3.71	(1.29, 10.7)	0.02	3.58	(1.15, 11.1)	0.03
**Start of sexual activity**							
15–18	19	0.52	(0.20, 1.37)	0.19			
> 18	10	*Ref*					
**Any symptom reported**							
No	47	*Ref*					
Yes	21	0.98	(0.54, 1.76)	0.94			

Odds ratios (OR) and 95% confidence intervals (CI) were calculated using univariate and multivariate logistic regression

## Discussion

In this study population, we found a period prevalence of HPV infection of 56% (Chachis 53% and Afro-Ecuadorian 57%). This value is comparable with the study conducted by Paez et al. [34], where women with normal and precancerous cervical lesions from rural areas of Ecuador were included. Similar values have also been reported in women from rural communities in other South American countries such as Brazil (53.3%) and Argentina (51.6%) [35, 36]. Surprisingly, HPV-58 was the most common HR-HPV, unlike other studies where HPV-16 is most prevalent [16, 28, 37, 38]. In Ecuador, HPV-58 was reported as the second genotype most frequently associated with cancer [16]. On the other hand, HPV-18 represented only 1% of infections, as in previous reports from Ecuador [23, 37, 38]. The same phenomenon has been described in other South American countries [39–41]. HPV-16 (20%) was the most prevalent in Afro-Ecuadorian women whereas HPV-58 (30%) in Chachis women which is consistent with other studies conducted in Ecuador [16, 23]. It is known that the prevalence of HR-HPV genotypes differs from one human population to another [8, 9]. Logistic and sociocultural variables could also be related to the presence of certain HPV types in the studied ethnic groups [22, 42, 43]. Although Chachis and Afro-Ecuadorian women are found in the same region, each group inhabit specific geographic areas that are difficult to access. This isolation may influence the dynamics of the circulation of HPV genotypes as suggested by Batista et al. [44]. In addition, the difference of host genetic background could contribute to the prevalence of a specific HR-HPV genotype. In particular, the HLAs alleles may play an important role in determining which HPV variant will clear or persist and progress to cervical cancer [45, 46].

It is important to highlight the presence of HR-HPV 68 and 39 which were described in lower rates in other Ecuadorian studies [16, 47–49]. HPV-68 was found mainly in co-infections, as described by others [16, 50–52]. Interestingly, the high HPV-39 prevalence has also been observed in women from Bahía, Brazil [37], and urban women from China [53]. Also, we found a prevalence of HPV-52 (6%) similar to data from East Asia where HPV-52 and -58 are more prevalent compared to worldwide [54, 55]. Most infections with low-risk genotypes (6, 11, and 43) were co-infected with HR-HPV. Something similar has been found in other studies where the prevalence of co-infections is greater than 50% [16, 56]. Therefore, knowledge of the regional prevalence of HPV genotypes is essential for the development of effective vaccination as well as preventative screening strategies against cervical cancer.

All HPV-16 sequences found in this study belonged to the European lineage regardless its ethnical origin which is in accordance with the previously published investigation in Ecuador and other rural populations in Latin America [16, 17, 28, 57, 58]. This lineage tends to be the least carcinogenic and persistent in comparison to the non-European variants, which show increased risk of developing cancerous lesions [12, 13]. All HPV-16 sequences grouped indistinctly into A1 and A4 sublineages. No specific polymorphisms were found in our sequences when compared to the HPV-16 European prototype, and they were similar to previous studies in Ecuadorian mestizo women with HPV-16 positive infection [16]. Both HPV-58 sequences in this study belonged to the A2 sublineage, which is the most common variant around the world [59] and has a lower risk of disease progression than A3 and A1 sublineages [13, 14, 60]. Our findings on HPV-16 and HPV-58 showed no difference between ethnicity, Chachi, Afro-Ecuadorian, and mestizo sequences. One SNP (G6641A, synonymous mutation) was found in one sequence from an Afro-Ecuadorian sample, and it has been previously described [61, 62].

We identified two risk factors that influenced HPV infection in this population: number of life sexual partners and community access by road (vs. river). Several studies have previously reported that higher number of sexual partners increases the risk of HPV infection and co-infections [63, 64]. A study in Mexico with 115,651 participants found that women with 6 or more lifetime sexual partner had twice the prevalence of HR-HPV infection than women with 1–5 partners [65]. In our smaller study of women from remote communities, we also found that having more than one lifetime sexual partner more than doubled the odds of HR-HPV infection after adjusting for other covariates. On the other hand, we found that women living in communities only accessible by river had a third of the odds of HR-HPV infection than women in areas more readily accessible by road. In isolated communities with access only by river, the flow of HPV-infected individuals might be restricted and, therefore, the probability of HPV infection might be lower. Similarly, Eisenberg et al. (2006) [66] evaluated the impact of road construction in this region on the epidemiology of diarrheal diseases. They noted higher infection rates of intestinal pathogens in communities with road access than in remote communities with only river access.

Much less has been discussed on the association between road accessibility and prevalence of HPV in remote communities. Notably, a study of HPV and other sexually transmitted infections in rural communities in Bolivia found no differences in the prevalence of HR-HPV infection between women from large towns, small towns, and villages [22]. Meanwhile, a different study among Amerindians from the Venezuelan Amazon reported an upward trend of the prevalence of any HPV as the urbanicity of communities increased [67]. The study also reported lower HPV diversity among more isolated Amerindians suggesting that geographic barriers may influence the viral genetic pool in communities that are harder to reach.

This study has limitations to note. Interviewer bias may have been introduced because participants did not complete surveys on their own but were assisted by a team member. As was previously mentioned, this was done to accommodate different cultural traditions and literacy levels. To minimize its impact, all team members were well trained in data collection methods and instructed to use a neutral nonjudgmental tone with all participants. We did not provide a specific definition of what a sexual partner or sexual activity entailed, which allowed participants to answer these questions based on their own definitions. For example, some may have had sexual activity including oral sex, but since it did not include penetration, they may not consider that as sexual activity or the other person as a sexual partner [68]. Additionally, this study included participants younger than 30 years of age and as young as 14, which is inconsistent with most current guidelines and may lead to false positive cases [69, 70]. Data for this study was collected before national and international guidelines were developed and when much less was known about the physiopathology of HPV. Currently, some debate still remains about the potential benefits of screening women younger than 30 [71]. Our small sample size may limit generalizability of findings, including populations in northwestern Ecuador. However, this cross-sectional study did not aim to discuss prevalence of HPV across the population of the area but to examine its prevalence in specific minoritized communities of Ecuador who mainly reside in the province of Esmeraldas.

Finally, our study contributes to a better understanding of HPV genotype distribution within Chachis and Afro-Ecuadorian populations that have been underrepresented in HPV research. The identification of HPV-58 as the most prevalent high-risk genotype provides valuable information that can be useful for vaccination strategies and screening programs in these populations.

## Conclusions

This study provides a baseline knowledge about the prevalence and distribution of HPV genotypes in the northwestern coastal of Ecuador. Prevalence of HPV in this region was higher compared to previous studies conducted in other regions of Ecuador. The most frequent genotypes were HPV-58, -16, -68, and -39. Studies of HPV prevalence are especially relevant in remote communities where access to health care is very limited. The distribution of the HPV genotypes varied according to the ethnic group. Most results were consistent with previous studies in Ecuador suggesting that geographic location is an important predictor of genotype type. Differences in the frequency of HPV genotypes in Amerindian and Afro populations should be further investigated. Conducting this study in remote populations of northwestern Ecuador represents a pioneering effort aimed at assessing the prevalence of HPV and circulating genotypes, with the goal of combating cervical cancer disparities. Notably, Ecuador lacked a national HPV vaccination plan during the study period, whose implementation began in 2014 using the bivalent vaccine (Cervarix®). However, as this article highlights, the vaccine may offer limited protection among communities where other genotypes circulate. An updated assessment in a post-vaccine era could shed light on the actual efficacy of this strategy among remote communities.

## Data Availability

The data and materials produced and examined in this study are not accessible to the public because of confidentiality agreements. Nonetheless, reasonable requests for access will be evaluated individually. Inquiries for access to the confidential data should be sent to the corresponding author at szapata@usfq.edu.ec. Each request will be assessed by the institutional ethics committee to ensure adherence to privacy and confidentiality guidelines.
